# A Cross-Sectional Survey Exploring Australian Pharmacists’ and Students’ Management of Common Oral Mucosal Diseases

**DOI:** 10.3390/pharmacy11050139

**Published:** 2023-09-01

**Authors:** Meng-Wong Taing, Joshua Choong, Vijayaprakash Suppiah, Sarira El-Den, Joon Soo Park, Michael McCullough, Leanne Teoh

**Affiliations:** 1 School of Pharmacy, The University of Queensland, Brisbane, QLD 4102, Australia; j.choong@uq.net.au; 2 Clinical and Health Sciences, University of South Australia, Adelaide, SA 5001, Australia; vijay.suppiah@unisa.edu.au; 3 School of Pharmacy, Faculty of Medicine and Health, The University of Sydney, Sydney, NSW 2006, Australia; sarira.el-den@sydney.edu.au; 4 UWA Dental School, The University of Western Australia, Nedlands, WA 6009, Australia; alex.park@uwa.edu.au; 5 Melbourne Dental School, University of Melbourne, Carlton, VIC 3053, Australia; m.mccullough@unimelb.edu.au (M.M.); leanne.teoh@unimelb.edu.au (L.T.)

**Keywords:** pharmacists, case vignettes, pharmacy students, oral mucosal diseases, oral health

## Abstract

Background: Oral mucosal conditions are commonly experienced in the general population and can have a negative impact on one’s quality of life. This study evaluated the ability of Australian pharmacists and final-year pharmacy students to recognise and manage these common oral mucosal diseases through the use of case vignettes. Methods: Australian pharmacists and final-year pharmacy students were invited through social media, university learning management systems, or email to complete an online questionnaire consisting of six case vignettes covering topics relating to common oral mucosal presentations. Results: A total of 65 pharmacists and 78 students completed the questionnaire. More than 50% of the participants reported having seen all types of oral mucosal presentations, except for denture stomatitis, in their practice. The provision of best practice recommendations was reported by only 14%, 15%, 8%, and 6% of the participants for geographic tongue, hairy tongue, angular cheilitis, and denture-associated stomatitis, respectively, whereas 82% offered an appropriate anti-viral treatment for cold sore and 33% provided the best practice recommendations for oral thrush. Conclusion: This study emphasised the importance of further developing and integrating best practice oral healthcare training programs specifically tailored to the Australian pharmacy profession.

## 1. Introduction

Good oral health contributes to overall health and wellbeing [1,2]. Poor oral health, on the other hand, is associated with oral pain, discomfort, and a reduced quality of life, which can affect an individual’s ability to eat, speak, and socialize [2,3]. In 2022, approximately 3.5 billion people worldwide were affected by oral diseases, and oral diseases are the most common chronic condition worldwide [4]. Between 2020 and 2021, less than half (48%) of Australians over the age of 15 visited a dental professional, while 1 in 9 (11%) children aged 5–14 years had never visited a dental practice [3]. Furthermore, nearly 67,000 hospitalisations in Australia between 2019 and 2020 were due to preventable dental conditions [3].

Community pharmacists in Australia are readily accessible to the public and are well positioned to recognise, prevent, and manage common oral health conditions [5]. With approximately 5800 community pharmacies equitably distributed throughout Australia and over 35,000 registered pharmacists [6,7], pharmacy professional services are mostly provided without charge or the need for an appointment; in outer regional and remote areas, pharmacists are often the first or only contact with a health professional [8,9]. The study by Taing et al. showed that more than 80% of Australian pharmacists were consulted for oral healthcare advice up to five times or more each week, with more than half being involved in identifying the signs and symptoms of oral health problems [10]. Therefore, it is crucial that community pharmacy staff are capable of appropriately identifying common oral conditions or issues and know when referral to a dental practitioner may be necessary [11].

Anecdotal reports from pharmacy academics in Queensland and New South Wales suggest that, on average, pharmacy students receive just two lectures during their entire program on topics related to oral health, and interprofessional education between pharmacy and dentistry students is slowly gaining momentum [10,12]. Australian practicing pharmacists are provided with continuing professional development in oral healthcare through seminars and magazine/journal articles [10]. Previously, case vignettes have been used to evaluate Australian pharmacy staff’s knowledge and management of various common oral conditions, including dental pain, gingivitis, dry mouth, non-healing mouth ulcers, and oral hygiene [5]. However, the findings by Taing et al. indicated that a significant proportion of pharmacists were not correctly identifying these conditions or providing the best practice care. These oral conditions are among the most commonly seen in community pharmacy, but do not include common mucosal diseases, such as cold sores, geographic tongue, hairy tongue, angular cheilitis, denture-associated erythematous stomatitis, and oral thrush [13,14]. These mucosal disorders commonly encountered in the Australian community are not associated with a significant morbidity and mortality, and can be managed in general practice [14]. 

Currently, no studies have assessed whether Australian pharmacy students or pharmacists can appropriately identify common mucosal diseases and have the knowledge to manage these conditions effectively. Therefore, the aim of this study was to evaluate the ability of Australian pharmacists and final-year pharmacy students to recognise and manage common oral mucosal diseases using case vignettes. This study also reports on the frequency of oral mucosal disease presentations encountered by participants during their practice. The findings from this study will provide an indication of how prepared pharmacists and pharmacy students are in identifying common oral mucosal disorders in practice and will inform future training requirements for delivering improved oral healthcare within Australian communities.

## 2. Materials and Methods

### 2.1. Design of Survey Questionnaire and Case Vignettes

Clinical or case vignettes are frequently used to evaluate the quality of care and depth of knowledge of health professionals [5,15]. This study employed 6 case vignettes to assess the ability of Australian pharmacists and final-year pharmacy students to manage common oral mucosal presentations [14], including cold sores (scenario A), geographic tongue (scenario B), hairy tongue (scenario C), angular cheilitis (scenario D), denture-associated erythematous stomatitis (scenario E), and oral thrush (scenario F). Final-year pharmacy students were selected because they are approaching the end of their undergraduate training and are likely to commence supervised pharmacy practice on a full-time basis, following which, a large proportion of them practice as community pharmacists [16].

The survey questionnaire and accompanying six case vignettes comprised a series of multiple choice, Likert scale, and open-response questions (see Supplementary Material A and B). For each vignette, a photographic representative of the condition and a brief description of the patient’s symptoms, medical history, and other relevant information were provided to improve the ecological validity. The cases were designed by an expert consensus within the multidisciplinary team, consisting of experts in oral medicine, dentistry, and pharmacy practice (authors MM, LT, and MWT), where the signs and symptoms representative of each condition and best practice recommendations were validated using evidence-based guidelines (see Table 1: summarised case vignettes and best practice recommendations) [11,14,17]. These guidelines were developed for non-dental practitioners and are relevant to patients of all ages and population groups [14,17]. The participants completed demographic questions at the beginning of the survey (Supplementary Material A) and the questionnaire items within all 6 case vignettes (Supplementary Material B). For each case vignette, participants were asked to rate their confidence in assessing and managing the condition, identify the presentation, provide treatment advice, list resources used to assist and advise, and on average how often they would see these types of oral mucosal presentations each week in their practice.

The case vignettes and demographic questionnaire items were piloted by practicing pharmacists (n = 4) and final-year pharmacy students (n = 5). Their feedback informed changes in phrasing and formatting to increase clarity. The final case vignettes, along with the demographic questionnaire items, were uploaded onto the online survey platform checkbox. The case vignettes were identical for the pharmacists and final-year pharmacy students, with slight differences in the demographic questionnaire items. (See Supplementary Material A and B) 

### 2.2. Participant Recruitment

The Australian practicing pharmacists (intern and registered), as well as the final-year pharmacy students, were recruited via convenience sampling between the months of May and August 2022. To recruit the final-year pharmacy students, an online advertisement containing the survey link/QR code was posted on final-year university learning management systems (LMS) websites and/or sent directly to student email accounts from final-year course convenors. To enhance the representation of final-year pharmacy students within Australia, four pharmacy schools from different states participated in the study: the University of Western Australia (UWA), the University of Sydney (USyd), the University of Queensland (UQ), and the University of South Australia (UniSA). The survey advertisement was distributed four times, at approximately 2–4 week intervals, by each university pharmacy program provider to encourage recruitment. The surveys were closed approximately 12 weeks after the first announcement. Final-year pharmacy students recruited at UQ were also provided with two extra reminders via a social media Facebook group (UQ Pharmacy Class of 2022), which comprised the majority of these final-year UQ pharmacy students. As an incentive to participate, the final-year UQ pharmacy students were provided with an AUD 5 coffee voucher for their participation. The students who completed the survey from other universities were included in a draw to win one of five AUD 50 eGift vouchers.

The pharmacists (intern and registered) were recruited nationally by posting the survey advertisement with the survey link/QR code six times via the Pharmaceutical Society of Australia’s Early Career Pharmacist social media Facebook page (ECP FBP). The ECP FBP has over 12,400 members, approximately one third of the total registered pharmacists in Australia, with the majority being pharmacists or interns within their first ten years of graduating. The ECP FBP is open to all pharmacists, with the goal of providing a space for the discussion of contemporary issues affecting pharmacists, and it is a frequently used platform for recruiting registered Australian pharmacists for research. Similar to recruiting pharmacy students, the survey advertisement containing the link/QR code was distributed four times at approximately 2–4 week intervals, and then an additional 2 rounds were conducted to improve response rates. Similar to the students, the pharmacists were also offered a chance to win one of ten AUD 50 eGift vouchers for completing the survey. 

### 2.3. Data Analysis

After the survey closure, coding templates were created for each case vignette to categorise the participant responses for the open-ended questions. The responses were coded as categorical variables within Microsoft Excel for Microsoft 365 (Washington, DC, USA) and SPSS v26 (SPSS Inc., Chicago, IL, USA) to enable a descriptive analysis. For the open-ended questions, the responses were coded as binary entries (correct [1] or incorrect [0]) within the coding templates. Pharmacist interns were categorised as pharmacists for analysis purposes, as their training is closely aligned with pharmacists. Students who reported practicing in pharmacy (68 out of 76 student responses) were included in the analysis relating to the average weekly frequency consulted for each presentation. Data from incomplete participant responses were excluded from the analyses.

To determine whether differences existed between the pharmacy students’ and pharmacists’ responses, Pearson’s chi-squared test was used. Fisher’s exact test was applied when the expected responses per cell were fewer than 5 [18]. Adjusted standardised residuals were used to identify cells with significantly larger/smaller counts than expected if the variables were independent in contingency tables larger than 2 × 2. The significance level was set at *p* < 0.05. The study was approved by the University of Queensland’s Human Research Ethics Committee (approval no. HE002431, date of approval 14 April 2022).

## 3. Results

### 3.1. Demographic Characteristics

A total of 390 case vignettes were completed by the Australian pharmacists, while the pharmacy students completed 456 case vignettes. Each participant completed 6 clinical cases, resulting in 65 survey completions by the pharmacists and 76 by the pharmacy students. The response rate represented less than 1% of the members of the ECP FBP, and 14% of the enrolled final-year pharmacy students at the four Australian universities. Table 2 presents the demographic characteristics of the pharmacists and pharmacy students. The pharmacists worked a median of 38 h per week (IQR 3–58 h), while the students worked a median of 15 h per week (IQR 5–30 h). The demographic statistics for the pharmacists related to state/territory of practice, location of pharmacy (degree of remoteness), and gender, and were largely comparable with the Australian national statistics [7,19]. However, the majority of the pharmacists (87%) participating in this study were younger, aged between 20 and 39 years, compared to the 59% reported nationally. National Australian pharmacy student demographic statistics were not available, and hence, a comparison with our sample student cohort could not be made.

### 3.2. Case Vignettes

#### 3.2.1. Recognising the Presentation

Almost all the practicing pharmacists and students appropriately identified the cold sore and oral thrush presentations (Case A and F, respectively, Table 3). However, only about half of the participants (44%; 62/141) correctly identified angular cheilitis (case D), with the pharmacists being more likely to identify the condition compared to students (*p* < 0.0001). Less than 20% of the participants correctly identified the geographic tongue and denture stomatitis presentations (case B and E, respectively), and almost all the participants were unable to identify the hairy tongue presentation (case C). For geographic and hairy tongue, the majority of participants believed the presentation was oral thrush (57%; 80/141 and 72%; 102/141, respectively). For angular cheilitis, the presentation was commonly misidentified as dry/cracked lips (12%; 17/141) or the respondents were unsure (19%; 27/141). Most participants (72%; 101/141) were unsure of the denture stomatitis presentation.

#### 3.2.2. Management

Table 4 describes whether the final-year pharmacy students and practicing pharmacists provided the best practice management recommendations for the six case vignettes, and for the students, whether they would refer to their supervising pharmacist.

For case A (cold sore), 82% (115/141) recommended the best practice of providing acyclovir 5% cream or famciclovir 1500 mg orally. However, 9% (7/76) of the students and 20% (13/65) of pharmacists recommended Virasolve^®^, a topical cream containing antiviral (idoxuridine) that is registered by the Therapeutic Goods Administration for the symptomatic relief of cold sores. However, it is not listed for the management of cold sores within the Therapeutic Guidelines (TG) [14].

For case B (geographic tongue), only 13% (19/141) of the participants recommended the best practice (Table 4). Approximately 20% of the pharmacists (18/65) and students (13/76) referred the patient to the dentist or doctor, and more than half of the pharmacists (56%; 36/65) and 41% (31/76) of the students inappropriately recommended an oral antifungal treatment (miconazole or nystatin).

For case C (hairy tongue), only 15% (21/141) of the respondents recommended the best practice (Table 4). The majority of the students and pharmacists (63% (48/76) and 62% (40/65), respectively) inappropriately recommended an antifungal agent (nystatin drops or miconazole gel). Approximately one in five pharmacists (13/65) and students (12/76) referred the patient to the dentist or doctor, and only three students would refer the case to the pharmacist.

For case D (angular cheilitis), only 8% (11/141) of the participants recommended the best practice (Table 4). The pharmacists and students commonly recommended the use of a moisturiser or lip balm (29% (19/65) pharmacists vs. 30% (23/76) students).

For case E (denture-associated erythematous stomatitis), only 6% (8/141) of the participants recommended the best practice (Table 4). A common, non-evidence-based recommendation included the provision of products for pain (16% (12/76) vs. 15% (10/65) for students and pharmacists, respectively).

For case F (oral thrush), only 33% (46/141) of the participants recommended the best practice (Table 4). Approximately one in ten pharmacists (7/65) and students (9/76) provided oral hygiene advice, and a small proportion of the respondents were unsure (5% (3/65) vs. 8% (6/76) of pharmacists and students, respectively).

#### 3.2.3. Confidence

Overall, the pharmacists reported being marginally more confident (SA/A) in their ability to diagnose and manage the case vignettes compared to the pharmacy students (53% (208/390) pharmacists and 42% (193/456) students, respectively, see Table 5). An analysis of the pharmacist/student confidence versus their ability to identify and appropriately manage the individual case vignettes is presented below.

For case A (cold sore), the pharmacists who self-reported being confident (SA/A) were more likely to correctly identify the condition as cold sore compared to those reporting N/D/SD (100% vs. 81.8%, respectively, *p* = 0.026). However, no associations were found between self-reported confidence (SA/A vs. N/D/SD) and the provision of best practice recommendations.

For case B (geographic tongue), the pharmacists and students self-reporting being confident (SA/A) were less likely to identify the condition as geographic tongue compared to those reporting N/D/SD (3.7% vs. 30.6%, respectively, for students, *p* = 0.007, and 10.5% vs. 33.3%, respectively, for pharmacists, *p* = 0.031). The pharmacists reporting higher levels of confidence (SA/A) were also less likely to provide the best practice recommendations for geographic tongue compared to those reporting N/D/SD (10.5% vs. 33.3%, respectively, *p* = 0.031).

For case C (hairy tongue), no significant associations were found between pharmacist/student self-reported confidence (SA/A vs. N/D/SD) and their ability to appropriately identify the condition or provide the best practice recommendations.

For case D (angular cheilitis), the pharmacists and students who self-reported being confident (SA/A) were more likely to identify the condition as angular cheilitis compared to those reporting N/D/SD (47.8% vs. 20.8%, respectively, for students, *p* = 0.027, and 77.8% vs. 41.4%, respectively, for pharmacists, *p* = 0.004). No significant associations were found between pharmacist/student self-reported confidence (SA/A vs. N/D/SD) and their ability to provide the best practice recommendations.

For case E (denture-associated erythematous stomatitis), the pharmacists who self-reported as being confident (SA/A) were more likely to identify the condition as denture-associated erythematous stomatitis compared to those reporting N/D/SD (40% vs. 5%, respectively, *p* = 0.044). No significant associations were found between pharmacist/student self-reported level of confidence (SA/A vs. N/D/SD) and their ability to provide the best practice recommendations.

For case F (oral thrush), the pharmacists and students who self-reported as being confident (SA/A) in their ability to manage the condition were more likely to correctly identify the condition as oral thrush compared to those reporting N/D/SD (95.3% vs. 63.6%, respectively, *p* = 0.002 and 93% vs. 69.7%, *p* = 0.012 for pharmacists and students, respectively). The pharmacists reporting higher confidence were also more likely to provide the best practice recommendations for oral thrush compared to those reporting N/D/SD (48.8% vs. 18.2%, respectively, *p* = 0.03).

#### 3.2.4. Resources Used to Assist

In all vignettes, 21% (97/456) of the students compared to 8% (32/390) of the pharmacists used resources to answer the cases. The proportion of students using resources ranged from 14% (11/76) for oral thrush and cold sore to 34% (26/76) for geographic tongue, while the proportion for pharmacists ranged from 3% (2/65) for oral thrush to 14% (9/65) for denture stomatitis. A variety of resources, including the Australian Medicines Handbook, Monthly Index of Medical Specialities, TG, and online internet searches (Google, DermNet, WebMD and Mayo Clinic), were referred to by the study participants.

#### 3.2.5. Frequency of Presentations

Most of the practicing pharmacists and students (i.e., at least 50%) reported encountering all types of oral mucosal presentations, except for denture stomatitis, either less than once/week or more frequently (Table 6). The denture-stomatitis-type presentations were encountered least frequency in practice, with 65% (87/133) of the respondents never having seen this in practice.

## 4. Discussion

This is the first study to assess the ability of Australian pharmacists and final-year pharmacy students in managing six common oral mucosal conditions, including cold sore, geographic tongue, hairy tongue, angular cheilitis, denture-associated erythematous stomatitis, and oral thrush. The majority of the practicing pharmacists and students reported seeing all types of oral mucosal presentations, except for denture stomatitis, either less than once/week or more. Using case vignettes, most of the practicing pharmacists and students were able to appropriately identify the cold sore and oral thrush presentations (97% and 84%, respectively) and most recommended the appropriate anti-viral treatments for cold sores. However, 15% or less of the participants provided the best practice advice for the geographic tongue, hairy tongue, angular cheilitis, and denture-associated stomatitis presentations (14%, 15%, 8%, and 6%, respectively). This contrast in ability to manage cold sore and oral thrush presentations compared to other oral mucosal issues may partly be explained by the availability of over-the-counter cold sore and oral thrush pharmacy medications in Australian community pharmacies. These conditions are therefore inherently taught in the Australian pharmacy curriculum and supported by pharmacy practice resources [20,21,22].

A concerning finding in this study was a clear demonstration of poor differentiation skills between geographic tongue, hairy tongue, and oral thrush. The majority of the participants confused geographic and hairy tongue as oral thrush (57% and 72%, respectively), which was also observed in how the participants managed these conditions—more than half inappropriately recommended topical oral antifungal treatments. Topical miconazole is widely available on the market for over-the-counter use, and although fungal resistance is currently relatively low, studies have shown *C. albicans* resistant strains with a geographical susceptibility lower in the UK compared to Italy [23]. Drawing parallels with antibiotic resistance, superficial mycoses are becoming more resistant to antifungal medications [23,24], which is consistent with the increased use of antimicrobial drugs and increased microbial resistance patterns [23]. There is a possibility that the indiscriminate provision of antifungal agents in the community may contribute to antifungal resistance patterns; however, more research is required to better elucidate the associations between antifungal resistance and its usage in clinical/community settings [24]. Collectively, the findings from this study indicate that, apart from cold sore presentations, there is a need to improve the training for both Australian pharmacists and undergraduate students in common oral mucosal presentations, particularly in how to differentiate between oral thrush and other similar presenting conditions such as hairy tongue.

This study found that the confidence levels varied widely among both the pharmacists and pharmacy students, ranging from 8 to 83% of pharmacists and 7 to 75% of student, respectively, reporting that they were confident in managing the presented oral mucosal conditions. However, the association between confidence and the ability to identify and provide the best practice recommendations in this study was mixed. Regardless of the case presented, confidence did not always translate into the appropriate identification and provision of the best practice recommendations. Interestingly, for geographic tongue, the pharmacists who reported higher confidence were significantly less likely to correctly identify and manage it appropriately. This highlights the limited usefulness of confidence as a predictor of the appropriate identification and management of common oral mucosal conditions in Australian pharmacy practice. These findings are consistent with a study by Taing et al., which showed that confidence among Australian pharmacists and assistants was not highly correlated with the appropriate management of related oral conditions, including tooth pain, gum problems, mouth ulcers, xerostomia, and oral health promotion [5]. Together, these studies demonstrate a disconnect between self-confidence and the ability to appropriately manage common oral health presentations in pharmacy practice. As a result, there is a need for the development and integration of oral healthcare training courses and resources tailored to the pharmacy profession, which could be embedded within undergraduate and postgraduate programs and continuing professional development courses. To be effective, the development of these training resources must be multi-disciplinary and include organisational partnerships between pharmacy and dentistry professions, universities, consumers, and government healthcare networks. These partners possess the skill sets and capabilities to create practical and effective resources that promote implementation [25]. Additionally, the effectiveness, feasibility, and patient acceptability/outcomes of these resources should be evaluated. The current lack of Australian pharmacy-specific resources to support oral healthcare [25] may explain why only a small proportion of the pharmacists and students referred to resources to support their decision making in the study.

Internationally, the implementation of oral healthcare interventions within pharmacy settings have been successful in providing patient benefits to communities. In the UK, an intervention study by Sturrock et al. showed positive responses from pharmacy staff and patients. The pharmacist-led intervention involved providing oral health promotion advice for dental caries, fluoride toothpaste, dietary advice, and appropriate care for teeth and dentures. Over 70% of 1069 patients who received the intervention reported that their oral health knowledge had improved significantly, and 65% of the participants stated that the way they cared for their teeth would change. Within this study, 64% of the participants agreed that pharmacies were “definitely” the right place to receive teeth/oral healthcare advice, while only 3% disagreed [26]. This study also demonstrated that pharmacy staff were able to connect with patients who had not visited dental practitioners for more than two years [26].

A limitation of this study is that the participant responses from the case vignettes may not have been entirely reflective of the respondents’ responses in an actual working environment [27]. Additionally, since the findings were self-reported, they may be subject to respondent recall and social desirability biases. In addition, while some conditions such as denture stomatitis were reported to be uncommonly presented, the participants may not have been aware or able to identify these conditions, and so these results may be partly reflective of the participants’ knowledge of the mucosal conditions rather than the true clinical presentations encountered. Moreover, the survey had low response rates (<1% for pharmacists and 14% for pharmacy students), not all Australian Universities were included in the student sample, and the characteristics of non-responders were not obtained. These low response rates may have been due to several reasons, including the length of the survey, the fact that pharmacists are an over-surveyed population, and the additional time pressures brought about by peak COVID infection rates during the survey period. Nonetheless, the sample of 65 pharmacists and 76 final-year pharmacy students would provide approximate population estimates with a 12% margin of error [28]. However, volunteer bias may have been present, as the respondents were mainly younger, and the responses may represent participants who had a greater interest in oral healthcare; hence, our findings likely represent the views of younger/early career pharmacists and those about to enter the Australian pharmacy workforce who have an interest in oral healthcare. The response rates from this study are comparable to many recent Australian pharmacy-practice-based surveys, which report similar responses from approximately <1% to 20% [29,30,31,32,33]. It is also worth noting that most participants practiced in community settings, which is reflective of the fact that minor ailments are mainly treated and triaged by community pharmacists. The strengths of the study included the collection of data from both final-year pharmacy students and practicing registered pharmacists, which provides valuable insight into the current provision of oral healthcare from students about to begin their internship training year and those already practicing in the industry. The case vignettes used in this study were also reflective of common oral mucosal conditions that may present in pharmacy settings, and the questionnaires were piloted for their content and face validity by a multidisciplinary team of dental and pharmacy academics, as well as practicing pharmacists and pharmacy students. Past studies have suggested that case vignette findings may be overestimated. For example, a recent simulated patient study showed that only 10% of pharmacy staff referred potentially cancerous oral lesions compared to a 50% referral rate reported in case vignettes [34]. Future simulated patient studies would be valuable in validating the findings of this study.

## 5. Conclusions

This study highlighted the need for improved training in common oral mucosal conditions for both Australian pharmacists and pharmacy students. While the pharmacists and students demonstrated strong abilities in recognising cold sore and oral thrush, they struggled with differentiating between conditions such as geographic tongue, hairy tongue, angular cheilitis, and denture-associated stomatitis, and appropriate management recommendations were not regularly provided. The confidence levels of the pharmacists and students varied widely, and there was a disconnect between self-confidence and the ability to appropriately manage common oral health presentations in pharmacy practice. The study demonstrated the need for the development and integration of oral healthcare training courses and resources tailored to the pharmacy profession, which could be embedded within undergraduate and postgraduate programs and continuing professional development courses. There is a need for multi-disciplinary collaboration to create practical and effective resources that promote implementation.

## Figures and Tables

**Table 1:** Case vignettes and best practice recommendations.

Case Vignettes	Best Practice Recommendations ^a^
**A: Cold sores (recurrent oral mucocutaneous herpes)**	
Janette, a 39-year-old woman, presents with small blisters on her lower lip, which she describes as feeling itchy and painful. They appeared a few days ago. She has hypertension and takes atenolol. She has no known allergies. She has been working extra hours lately and has been under stress at work. The blisters seem to appear when she feels run down.	Janette should be provided with oral or topical antiviral treatment ^b^.
**B: Geographic tongue**	
Joseph, a 26-year-old man, would like some advice about his tongue that he describes to you as asymptomatic. He does not have any current medical conditions, take any other medicines, and has no known allergies or adverse reactions to medications.	Joseph requires a correct diagnosis of his condition as geographic tongue and reassurance that the condition is benign.
**C: Hairy tongue**	
Megan (29 years old) comes into your pharmacy and would like some advice about her tongue. She describes that she has changed her diet recently by replacing most of her diet with weight loss shakes (as she is trying to lose weight). She does not have any current medical conditions, take any other medicines, and has no known allergies or adverse reactions to medications.	Megan requires identification of the cause of her presentation being due to a lack of coarse foods in her new diet and addressing this, e.g., increasing her intake of coarse foods, or suggesting at least one of the following strategies: use of sodium bicarbonate mouthwash, brushing her tongue with a toothbrush, and/or practicing optimal oral hygiene ^c^.
**D: Angular cheilitis**	
Doris, a 78-year-old lady, comes into the pharmacy complaining of red painful sores on each side of her mouth, which started a few days ago. She has hypertension and is taking irbesartan/hydrochlorothiazide, and has depression, for which she takes sertraline. She also wears dentures and has no known allergies.	Doris requires referral to a dental practitioner for a dental review to assess for any dental/denture-related issues (e.g., reduced vertical dimension); enquiry about dry mouth or referral to a dentist for dry mouth assessment; and treatment with a topical antifungal ^d^.
**E: Denture-associated erythematous stomatitis**	
Mrs AB presents with a red patch on her palate, wears a denture, and requests a topical antifungal medicine. She is a non-smoker, does not use steroid inhalers, and does not have any immune compromise. She does not have any current medical conditions, take any other medications, and has no known allergies or adverse reactions to medications.	Mrs AB should be referred to a dental practitioner to assess her denture fit. She should also practice optimal oral ^c^ and denture hygiene ^e^.
**F: Oral thrush (pseudomembranous candidiasis)**	
Cody, a 34-year-old regular customer of your pharmacy, presents describing that he has noticed a creamy white appearance on the roof of his mouth. He says that he has wiped a bit of it off with his nails and it is a bit painful and also a bit red and raw. He has type 1 diabetes and uses insulin. He does not have allergies or adverse reactions to medications.	Cody should be referred to a doctor for an assessment of his diabetes (as poorly controlled diabetes can predispose for oral candidiasis). Cody should then be provided with optimal antifungal treatment for oral candidiasis ^f^.

^a^ Best practice recommendations based upon oral and dental therapeutic guidelines. ^b^ Either use of aciclovir 5% cream topically every 4 h for 5 days or 1500 mg oral famiciclovir as a single dose. ^c^ Oral hygiene can include: interdental cleaning (use of dental floss or interdental brushes once a day prior to brushing teeth), tooth and tongue cleaning (use of toothbrushes for 2 min twice a day with fluoride toothpaste, spitting out toothpaste, avoid rinsing mouth after brushing, and brushing tongue but not to brush or massage gums), mouthwash (avoid use of alcohol-containing mouthwashes, use of antiseptic mouthwashes, and use of anti-inflammatory and analgesic mouthwashes for symptomatic relief of some inflammatory oral mucosal diseases). ^d^ Either use of clotrimazole 1% cream twice a day for at least 14 days and continue treatment for 14 days after symptoms resolve or miconazole 2% cream twice a day for at least 14 days and continue treatment for 14 days after symptoms resolve. ^e^ Denture hygiene can include: cleaning dentures twice a day with warm water, mild soap and a toothbrush/denture brush/soft nail brush whilst avoiding cleaning with hot water, toothpaste, kitchen detergents, laundry bleaches, methylated spirits, and antiseptics or abrasives (unless advised by a dental practitioner). Clean gums and remaining teeth with soft toothbrush and toothpaste. Place dentures in a dry environment overnight after cleaning. ^f^ Optimal antifungal treatment for oral candidiasis include either: Miconazole 2% gel, 2.5 mL applied topically (then swallowed) 4 times daily after food for 7–14 days then continue for 7 days after symptoms resolve OR Nystatin liquid 100,000 IU/mL, 1 mL topically (then swallowed) 4 times a day after food for 7–14 days then continue for 2–3 days after symptoms resolve.

**Table 2:** Australian pharmacist and final-year pharmacy student demographic characteristics.

Respondent	Pharmacists	Respondent	Students
	n = 65		n = 76
**State/Territory**		**University**	
Queensland	18 (27.7%)	University of Southern Australia	19 (25.0%)
New South Wales	14 (21.5%)	University of Western Australia	8 (10.5%)
Victoria	17 (26.2%)	University of Sydney	22 (28.9%)
Tasmania	5 (7.7%)	University of Queensland	27 (35.5%)
South Australia	3 (4.6%)		
Western Australia	5 (7.7%)		
Northern Territory	1 (1.5%)		
Australian Capital Territory	2 (3.1%)		
**Pharmacist primary setting**		**Student workplace primary setting**	
Community Pharmacy	51 (78.5%)	Community Pharmacy	60 (78.9%)
Hospital Pharmacy	11 (16.9%)	Hospital Pharmacy	8 (10.5%)
No Current Pharmacy Employment	-	No Current Pharmacy Employment	8 (10.5%)
Other	3 (4.6%)	Other	-
**Location of Pharmacy (accessibility)**		**Location of Pharmacy (accessibility)**	
Highly accessible (Metropolitan area)	39 (60.0%)	Highly accessible (Metropolitan area)	60 (88.2%)
Accessible/Moderately accessible (Regional/Rural area)	24 (36.9%)	Accessible/Moderately accessible (Regional/Rural area)	8 (11.9%)
Remote/Very remote	2 (3.1%)	Remote/Very remote	-
**Age**		**Age**	
Under 20	-	Under 20	1 (1.3%)
20–29	30 (46.2%)	20–29	71 (93.4%)
30–39	27 (41.5%)	30–39	5 (6.6%)
40–49	7 (10.8%)	40–49	-
50–59	1 (1.5%)	50–59	-
60 and over	-	60 and over	-
**Gender**		**Gender**	
Female	52 (80.0%)	Female	52 (68.4%)
Male	11 (16.9%)	Male	24 (31.6%)
Other	2 (3.1%)	Other	-
**Post Graduate Qualifications**		**Post Graduate Qualifications**	
Yes	24 (36.9%)	Yes	4 (5.3%)
No	41 (63.1%)	No	72 (94.7%)
**Prior training/CPD in oral health**		**Oral health training in university**	
Yes	9 (13.8%)	Yes	42 (55.3%)
No	56 (86.2%)	No	34 (44.7%)
Main Role			
Employee pharmacist	45 (69.2%)		
Pharmacy manager	11 (16.9%)		
Pharmacy owner	6 (9.2%)		
Intern pharmacist	2 (3.1%)		
Other	1 (1.5%)		

**Table 3:** Recognising oral mucosal presentations.

		Correct Identification	
		Student	Pharmacist
Case	Presentation	n = 76	n = 65
A	Cold sore	74 (97%)	65 (97%)
B	Geographic tongue	16 (21%)	13 (20%)
C	Hairy tongue	1 (1%)	0 (0%)
D	Angular cheilitis	22 (29%)	40 (62%)
E	Denture stomatitis	40 (14%)	10 (15%)
F	Oral thrush	63 (83%)	55 (85%)

**Table 4:** Best practice oral health recommendations by pharmacists and final-year pharmacy students.

	Student	Pharmacist
**Case Vignette A: (Janette/Cold Sore)**	n = 76	n = 65
Provided best practice recommendation ^a^	64 (84.2%)	51 (78.5%)
Referral to pharmacist	0 (0%)	-
**Case Vignette B: (Joseph/Geographic Tongue)**		
Provided best practice recommendation ^b^	7 (9.2%)	12 (18.5%)
Referral to pharmacist	2 (3%)	-
**Case Vignette C: (Megan/Hairy Tongue)**		
Provided best practice recommendation/s ^c^	8 (10.5%)	13 (20%)
Identified change in diet as the cause/addressing this	8 (10.5%)	13 (20%)
Recommend sodium bicarbonate mouthwash	1 (1.3%)	0 (0%)
Brush tongue with toothbrush	6 (7.9%)	10 (15.4%)
Practice good oral hygiene	6 (7.9%)	12 (18.5%)
Referral to pharmacist	3 (4%)	-
**Case Vignette D: (Doris/Angular Cheilitis)**		
Provided best practice recommendations ^d^	6 (7.9%)	5 (7.7%)
Referred to dentist for denture-related issues	18 (23.7%)	23 (35.4%)
Enquired/referred regarding dry mouth	12 (15.8%)	13 (20%)
Topical antifungal therapy (clotrimazole/miconazole)	16 (21.1%)	26 (40%)
Referral to pharmacist	1 (1%)	-
**Case Vignette E: (Mrs Johnson/Denture-Associated Erythematous Stomatitis)**		
Provided best practice recommendations ^e^	5 (6.6%)	3 (4.6%)
Optimise denture hygiene	13 (17.1%)	8 (12.3%)
Referred to dentist for denture fit	11 (14.5%)	23 (35.4%)
Optimise oral hygiene	12 (15.8%)	12 (18.5%)
Referral to pharmacist	3 (4%)	-
**Case Vignette F: (Cody/Oral Thrush)**		
Provided best practice recommendations ^f^	21 (27.6%)	25 (38.5%)
Recommend assessment of diabetes	25 (32.9%)	30 (46.1%)
Miconazole oral gel/Nystatin	55 (72.4%)	49 (75.4%)
Referral to pharmacist	0 (0%)	-

^a^ Best practice recommendations for cold sores involve providing appropriate antiviral therapy. ^b^ Best practice recommendation for geographic tongue involve providing no pharmacological treatment, just reassurance that the condition is benign. ^c^ Best practice recommendation for hairy tongue involves either identifying the cause, which in this case is due to lack of coarse food/change in diet and addressing this, or suggesting at least one of the following strategies: use of sodium bicarbonate mouthwash, brushing tongue with toothbrush, or practicing good oral hygiene. ^d^ Best practice recommendation for angular cheilitis involves providing ALL 3 recommendations: referring to a dentist for a dental review to assess for any dental/denture-related issues AND enquiring or referral for assessment of dry mouth AND providing a topical antifungal. ^e^ Best practice recommendation for denture-associated erythematous stomatitis involves providing ALL 3 recommendations: optimising denture hygiene AND optimising oral hygiene AND referring to a dentist to assess denture-fit. ^f^ Best practice recommendation for oral thrush involves providing ALL 2 recommendations: recommending an assessment of his diabetes AND providing an appropriate antifungal therapy.

**Table 5:** Confidence in managing oral mucosal presentations.

		Confidence (SA/A)	
		Student	Pharmacist
Case	Presentation	n = 76	n = 65
A	Cold sore	57 (75%)	54 (83%)
B	Geographic tongue	27 (35%)	38 (58%)
C	Hairy tongue	38 (50%)	32 (49%)
D	Angular cheilitis	23 (30%)	36 (55%)
E	Denture stomatitis	5 (7%)	5 (8%)
F	Oral thrush	43 (57%)	43 (66%)

**Table 6:** Frequency of oral mucosal presentations.

		Frequency (Average/Week)					
		Never		Less than 1		1 or More	
		Student	Pharmacist	Student	Pharmacist	Student	Pharmacist
Case	Presentation	n = 68	n = 65	n = 68	n = 65	n = 68	n = 65
A	Cold sore	5 (7%)	9 (14%)	23 (34%)	14 (21%)	40 (59%)	42 (65%)
B	Geographic tongue	24 (35%)	17 (26%)	32 (47%)	24 (37%)	12 (18%)	24 (37%)
C	Hairy tongue	21 (31%)	20 (31%)	33 (49%)	24 (37%)	13 (20%)	21 (32%)
D	Angular cheilitis	32 (47%)	19 (29%)	22 (32%)	39 (60%)	14 (21%)	7 (11%)
E	Denture stomatitis	40 (59%)	47 (72%)	23 (34%)	16 (25%)	5 (7%)	2 (3%)
F	Oral thrush	25 (37%)	19 (29%)	23 (34%)	28 (43%)	20 (29%)	18 (28%)

## Data Availability

Data available on request due to ethical restrictions.

## Supplementary Materials

The following supporting information can be downloaded at: https://www.mdpi.com/article/10.3390/pharmacy11050139/s1, Supplementary Material A: Table S1. Non-UQ Student Questionnaire; Table S2. UQ Student Questionnaire; Table S3. Pharmacist Questionnaire, Supplementary Material B. Case Vignettes Provided to All Participants.
