# Supplementary material for: A Cross-Sectional Survey Exploring Australian Pharmacists’ and Students’ Management of Common Oral Mucosal Diseases
Source: Pharmacy (Basel). 2023 Sep 1;11(5):139. doi: 10.3390/pharmacy11050139 (PMC10514864; doi:10.3390/pharmacy11050139)
Supplement: Supplementary file 1 [file pharmacy-11-00139-s001.zip › pharmacy-2547447-supplementary.pdf]

Supplementary Material A) Questionnaires

Table S1) Non-UQ Student Questionnaire

1. Are you currently a final-year pharmacy student studying within Australia?

☐ No (If you answer no to this question, we greatly appreciate your time, however, we are only collecting responses from final-year Australian pharmacy students → directed to end of survey).
☐ Yes

2. Which University do you study at? (Please choose only one of the following)

☐ The University of Sydney ☐ The University of South Australia

☐ The University of Western Australia

3. Are you currently working in pharmacy?

☐ No (If you respond no, skip questions 4-6)
☐ Yes

4. Please indicate the setting where you do most of your work in.

☐ Community Pharmacy ☐ Hospital Pharmacy

☐ Other ____________________________

5. On average, how many hours do you practice in community pharmacy each week?

____

6. The pharmacy you do the majority of your work in is:

☐ Highly accessible (Metropolitan Area) ☐ Accessible / Moderately accessible (Regional/Rural Area)

☐ Remote/Very remote

7. Gender:

☐ Male ☐ Female ☐ Other ____________

8. What is your age?

☐ Under 20
☐ 20 - 29
☐ 30 - 39
☐ 40 - 49
☐ 50 - 59
☐ 60 or over

9. Do you have any post-graduate qualifications? If yes, please specify (no abbreviations please)

☐ No
☐ Yes

10. In your pharmacy program, have you received oral healthcare education/training?

☐ No (→ Start Section 2)
☐ Yes (→ Q11)
Please specify: _____________

11. How many hours of oral healthcare education/training have you received?

☐ Under 5
☐ 5 – 10
☐ 10 - 20
☐ 20 - 30
☐ 30 or over

Table S2) UQ Student Questionnaire

1. Are you currently a UQ final-year pharmacy student?

☐ No (If you answer no to this question, we greatly appreciate your time, however, we are only collecting responses from 4th year Australian pharmacy students → directed to end of survey).
☐ Yes

2. Are you currently working in pharmacy?

☐ No (If you respond no, skip questions 3-5)
☐ Yes

3. Please indicate the setting where you do most of your work in.

☐ Community Pharmacy ☐ Hospital Pharmacy

☐ Other ____________________________

4. On average, how many hours do you practice in community pharmacy each week?

____

5. The pharmacy you do the majority of your work in is:

☐ Highly accessible (Metropolitan Area)
☐ Accessible / Moderately accessible (Regional/Rural Area)
☐ Remote/Very remote
☐ Other _____________

6. Gender:

☐ Male ☐ Female ☐ Other ____________

7. What is your age?

☐ Under 20
☐ 20 - 29
☐ 30 - 39
☐ 40 - 49
☐ 50 - 59
☐ 60 or over

8. Do you have any post-graduate qualifications? If yes, please specify (no abbreviations please)

☐ No
☐ Yes

9. In your pharmacy program, have you received oral healthcare education/training?

☐ No (→ Start Section 2)
☐ Yes (→ Q10),
Please specify: _____________

10. How many hours of oral healthcare education/training have you received?

☐ Under 5
☐ 5 – 10
☐ 10 - 20
☐ 20 - 30
☐ 30 or over

Table S3): Pharmacist Questionnaire

1. Are you currently practicing as a pharmacist?

☐ No (we greatly appreciate your time, however we are only collecting responses from practicing pharmacists → directed to end of survey)
☐ Yes

2. In which Australian state or territory is your pharmacy located? (Please choose only one of the following)

☐ Queensland ☐ New South Wales ☐ Victoria

☐ South Australia ☐ Northern Territory ☐ Western Australia

☐ Australian Capital Territory ☐ Tasmania

3. Please indicate the setting where you do most of your work in.

☐ Community Pharmacy ☐ Hospital Pharmacy

☐ Other ____________________________

4. Are you the:

☐ Pharmacy owner ☐ Pharmacy manager ☐ Employee pharmacist

☐ Intern pharmacist ☐ Other ______________________________

5. The pharmacy you do the majority of your work in is:

☐ Highly accessible (Metropolitan Area) ☐ Accessible / Moderately accessible (Regional/Rural Area)

☐ Remote/Very remote

6. On average, how many hours do you practice in community pharmacy each week?

____

7. Gender:

☐ Male ☐ Female ☐ Other ____________

8. What is your age?

☐ Under 20
☐ 20 - 29
☐ 30 - 39
☐ 40 - 49
☐ 50 - 59

9. How many years of work experience as a pharmacist do you have?

☐ 1 – 3 years
☐ 3 – 5 years

☐ 5 – 10 years
☐ 10 – 20 years
☐ Over 20 years

10. Do you have any post-graduate qualifications?

☐ No
☐ Yes (Please specify - no abbreviations please)________________________

11. Within the past 5 years, have you received or completed any continuing professional development (CPD) resources in the area of oral health care?

☐ No
☐ Yes (Please describe)____________________

Supplementary Material B: Case vignettes provided to all participants

Case A

Scenario:
Janette, a 39-year-old woman, presents with small blisters on her lower lip which she describes as feeling itchy and painful. They appeared a few days ago. She has hypertension and takes atenolol. She has no known allergies. She has been working extra hours lately and has been under stress at work. The blisters seem to appear when she feels run down.

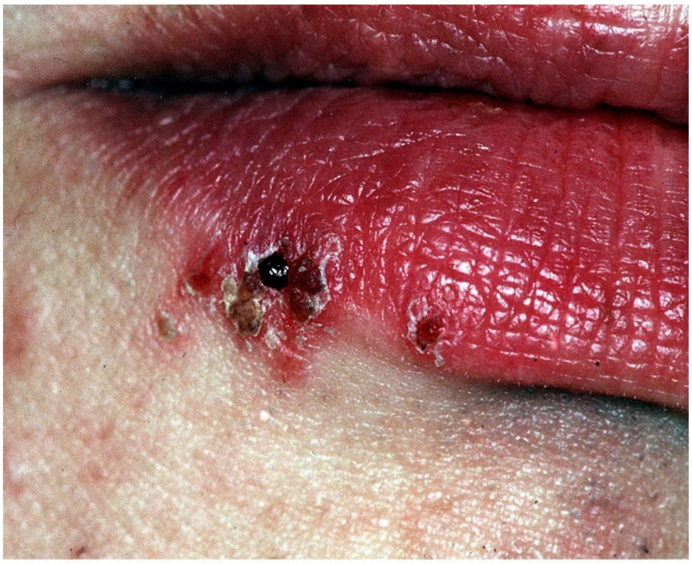

The next questions are designed to assess your confidence, awareness, and knowledge about how to manage Janette's symptoms.

1. **I am confident in my ability to appropriately manage Janette's presentation (Choose only one)**

- ☐ Strongly Agree
- ☐ Agree
- ☐ Neutral
- ☐ Disagree
- ☐ Strongly Disagree

2. **What do you identify the issue/presentation to be based on Janette's symptoms outlined in the scenario? (Open answer)**
3. **What other mucosal conditions could this be? (Open answer)**

4. **What treatment advice would you recommend for Janette? (Open answer)**

5. **Did you use any resources to answer this scenario?**

- ☐ Yes (please describe):___________________
- ☐ No

6. **At your main place of practice, on average how often would you be consulted for this type of presentation? N/A, Never, less than 1, 1, 2, 3, 4, 5 or 6+ consults/week.**

Case B
Scenario:
Joseph, a 26 year old man, would like some advice about his tongue that he describes to you as asymptomatic. He does not have any current medical conditions nor does he take any other medicines. He has no known allergies or adverse reactions to medications.

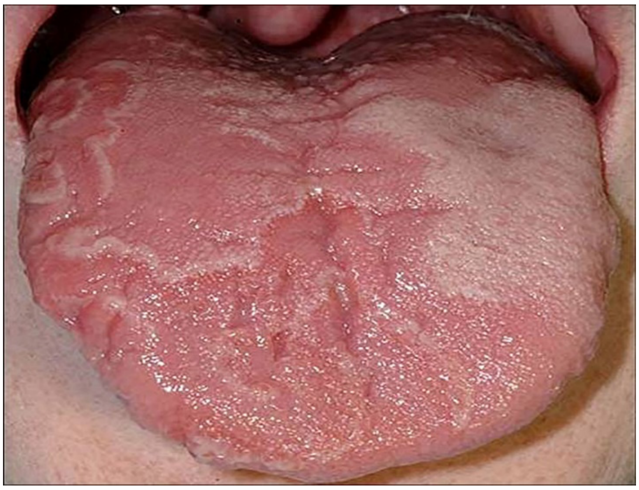

The next questions are designed to assess your confidence, awareness and knowledge about how to manage Joseph's symptoms.

1. **I am confident in my ability to appropriately manage Joseph's presentation (Choose only one)**

- ☐ Strongly Agree
- ☐ Agree
- ☐ Neutral
- ☐ Disagree
- ☐ Strongly Disagree

2. **What do you identify the issue/presentation to be based on Joseph's symptoms outlined in the scenario? (Open answer)**
3. **What other mucosal conditions could this be? (Open answer)**

4. **What treatment advice would you recommend for Joseph? (Open answer)**

5. **Did you use any resources to answer this scenario?**

- ☐ Yes (please describe):___________________
- ☐ No

6. **At your main place of practice, on average how often would you be consulted for this type of presentation? N/A, Never, less than 1, 1, 2, 3, 4, 5 or 6+ consults/week.**

Case C
Scenario:
Megan (29 years old) comes into your pharmacy and would like some advice about her tongue. She describes that she has changed her diet recently by replacing most of her diet with weight loss shakes (as she is trying to lose weight). She does not have any current medical conditions, take any other medicines and has no known allergies or adverse reactions to medications.

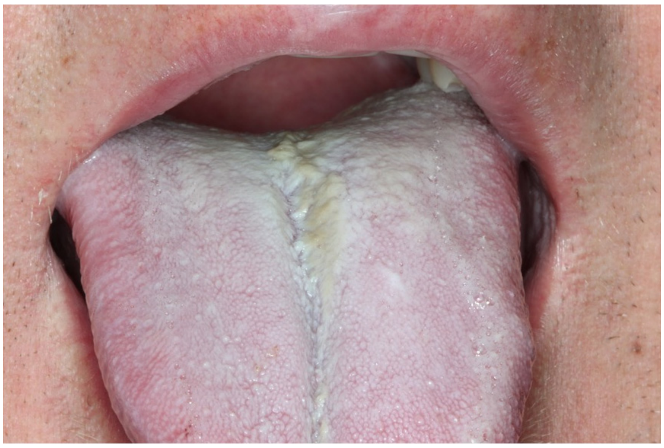

The next questions are designed to assess your confidence, awareness and knowledge about how to manage Megan's symptoms.

1. **I am confident in my ability to appropriately manage Megan's presentation (Choose only one)**

- ☐ Strongly Agree
- ☐ Agree
- ☐ Neutral
- ☐ Disagree
- ☐ Strongly Disagree

2. **What do you identify the issue/presentation to be based on Megan's symptoms outlined in the scenario? (Open answer)**
3. **What other mucosal conditions could this be? (Open answer)**

4. **What treatment advice would you recommend for Megan? (Open answer)**

5. **Did you use any resources to answer this scenario?**

- ☐ Yes (please describe):___________________
- ☐ No

6. **At your main place of practice, on average how often would you be consulted for this type of presentation? N/A, Never, less than 1, 1, 2, 3, 4, 5 or 6+ consults/week.**

Case D
Scenario:
Doris, a 78 year old lady comes into the pharmacy complaining of red painful sores on each side of her mouth which started a few days ago. She has hypertension and is taking irbesartan/hydrochlorothiazide. She also has depression for which she is taking sertraline. She also wears dentures and has no known allergies.

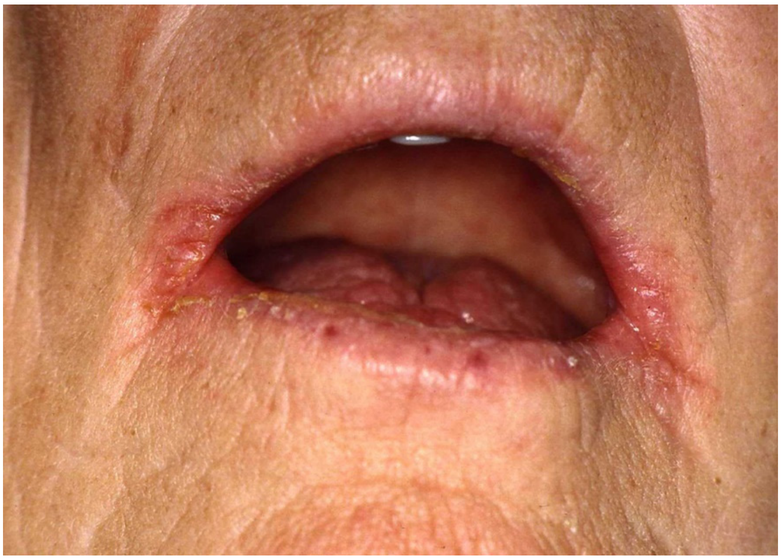

The next questions are designed to assess your confidence, awareness and knowledge about how to manage Doris's symptoms.

1. **I am confident in my ability to appropriately manage Doris's presentation (Choose only one)**

- ☐ Strongly Agree
- ☐ Agree
- ☐ Neutral
- ☐ Disagree
- ☐ Strongly Disagree

2. **What do you identify the issue/presentation to be based on Doris's symptoms outlined in the scenario? (Open answer)**
3. **What other mucosal conditions could this be? (Open answer)**

4. **What treatment advice would you recommend for Doris? (Open answer)**

5. **Did you use any resources to answer this scenario?**

- ☐ Yes (please describe):___________________
- ☐ No

6. **At your main place of practice, on average how often would you be consulted for this type of presentation? N/A, Never, less than 1, 1, 2, 3, 4, 5 or 6+ consults/week.**

Case E
Scenario:
Mrs Johnson presents with a red patch on her palate, wears a denture, and makes a request for a topical antifungal medicine. She is a non-smoker, does not use steroid inhalers and is not immunocompromised. She does not have any current medical conditions nor does she take any other medicines. She has no known allergies or adverse reactions to medications.

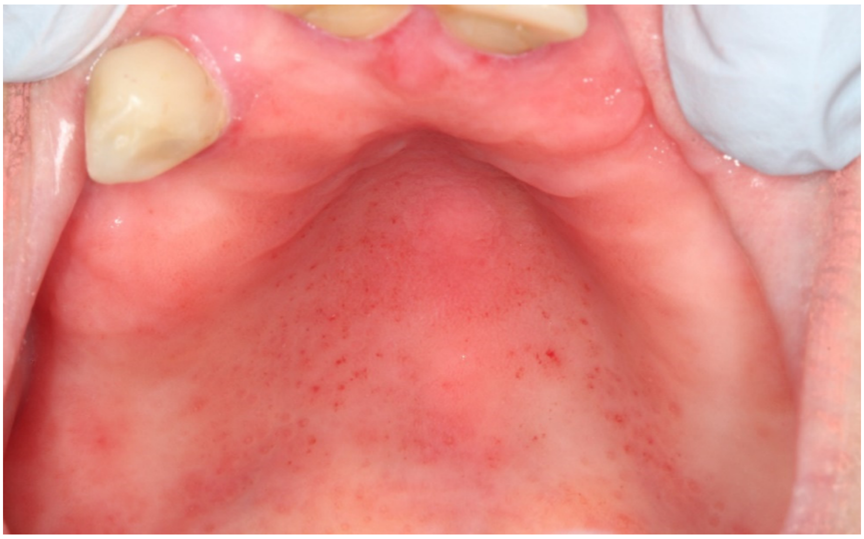

The next questions are designed to assess your confidence, awareness and knowledge about how to manage Mrs Johnson's symptoms.

1. **I am confident in my ability to appropriately manage Mrs Johnson's presentation (Choose only one)**

☐ Strongly Agree
☐ Agree
☐ Neutral
☐ Disagree
☐ Strongly Disagree

2. **What do you identify the issue/presentation to be based on Mrs Johnson's symptoms outlined in the scenario? (Open answer)**
3. **What other mucosal conditions could this be? (Open answer)**

4. **What treatment advice would you recommend for Mrs Johnson? (Open answer)**

5. **Did you use any resources to answer this scenario?**

☐ Yes (please describe):___________________
☐ No

6. **At your main place of practice, on average how often would you be consulted for this type of presentation? N/A, Never, less than 1, 1, 2, 3, 4, 5 or 6+ consults/week.**

Case F
Scenario:
Cody, a 34 year old regular customer of your pharmacy presents describing that he has noticed a creamy-white appearance in the roof of his mouth. He says that he has wiped a bit of it off with his nails. He also says it is painful, red and raw. He has Type 1 diabetes and uses insulin. He does not have allergies or adverse reactions to medications.

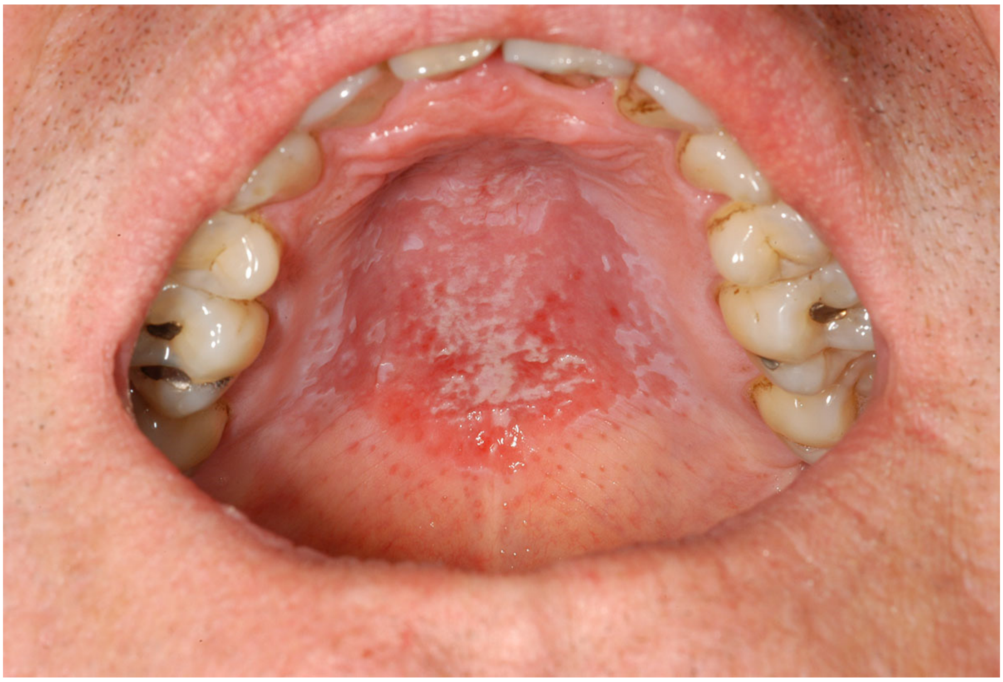

The next questions are designed to assess your confidence, awareness and knowledge about how to manage Cody's symptom.

1. **I am confident in my ability to appropriately manage Cody's presentation (Choose only one)**

- ☐ Strongly Agree
- ☐ Agree
- ☐ Neutral
- ☐ Disagree
- ☐ Strongly Disagree

2. **What do you identify the issue/presentation to be based on Cody's symptoms outlined in the scenario? (Open answer)**
3. **What other mucosal conditions could this be? (Open answer)**
4. **What treatment advice would you recommend for Cody? (Open answer)**
5. **Did you use any resources to answer this scenario?**

- ☐ Yes (please describe):___________________
- ☐ No

6. **At your main place of practice, on average how often would you be consulted for this type of presentation? N/A, Never, less than 1, 1, 2, 3, 4, 5 or 6+ consults/week.**
